# Association between egg consumption and cardiovascular disease events, diabetes and all-cause mortality

**DOI:** 10.1007/s00394-017-1566-0

**Published:** 2017-11-02

**Authors:** Jing Guo, Ditte A. Hobbs, John R. Cockcroft, Peter C. Elwood, Janet E. Pickering, Julie A. Lovegrove, David I. Givens

**Affiliations:** 1 Institute for Food, Nutrition and Health, University of Reading, Reading, RG6 6AR UK; 2 Hugh Sinclair Unit of Human Nutrition, University of Reading, Reading, RG6 6AR UK; 3 Institute for Cardiovascular and Metabolic Research, University of Reading, Reading, RG6 6AR UK; 4 Wales Heart Research Institute, Cardiff University, Cardiff, UK; 5 Department of Primary Care and Public Health, Cardiff University, Cardiff, UK

**Keywords:** Eggs, Cardiovascular disease, Type 2 diabetes, Impaired glucose tolerance, All-cause mortality

## Abstract

**Purpose:**

The association between egg consumption and cardiovascular disease (CVD) or type 2 diabetes (T2D) remains controversial. We investigated the association between egg consumption and risk of CVD (primary outcome), T2D and mortality in the Caerphilly prospective cohort study (CAPS) and National Diet and Nutritional Survey (NDNS).

**Methods:**

CAPS included 2512 men aged 45–59 years (1979–1983). Dietary intake, disease incidence and mortality were updated at 5-year intervals. NDNS included 754 adults aged 19–64 years from 2008 to 2012.

**Results:**

Men free of CVD (*n* = 1781) were followed up for a mean of 22.8 years, egg consumption was not associated with new incidence of CVD (*n* = 715), mortality (*n* = 1028) or T2D (*n* = 120). When stroke (*n* = 248), MI (*n* = 477), heart failure (*n* = 201) were investigated separately, no associations between egg consumption and stroke and MI were identified, however, increased risk of stroke in subjects with T2D and/or impaired glucose tolerance (IGT, fasting plasma glucose ≥ 6.1 mmol/L), adjusted hazard ratios (95% CI) were 1.0 (reference), 1.09 (0.41, 2.88), 0.96 (0.37, 2.50), 1.39 (0.54, 3.56) and 2.87 (1.13, 7.27) for egg intake (*n*) of 0 ≤ *n* ≤ 1, 1 < *n* ≤ 2, 2 < *n* ≤ 3, 3 < *n* < 5, and *n* ≥ 5 eggs/wk, respectively (*P* = 0.01). In addition, cross-sectional analyses revealed that higher egg consumption was significantly associated with elevated fasting glucose in those with T2D and/or IGT (CAPS: baseline *P* = 0.02 and 5-year *P* = 0.04; NDNS: *P* = 0.05).

**Conclusions:**

Higher egg consumption was associated with higher blood glucose in subjects with T2D and/or IGT. The increased incidence of stroke with higher egg consumption among T2D and/or IGT sub-group warrants further investigation.

**Electronic supplementary material:**

The online version of this article (doi:10.1007/s00394-017-1566-0) contains supplementary material, which is available to authorized users.

## Introduction

Cardiovascular diseases (CVD) are still the leading cause of morbidity and mortality, and the prevalence of type 2 diabetes mellitus (T2D) is also increasing globally. Diet plays an important role in prevention and management of both CVD [1] and T2D [2]. Eggs are a good source of a number of nutrients in the UK diet such as vitamin D, selenium, vitamin K and choline as well as high quality protein [3]. However, eggs also contain relatively high amounts of dietary cholesterol (350 mg/100 g) [4], which has been associated with impaired glucose metabolism [5] and increased inflammation [6] in animal models and with elevated fasting glucose in humans [7]. Furthermore, meta-analyses of intervention studies have shown that high consumption of dietary cholesterol increases serum total, LDL- and HDL-cholesterol concentrations, as well as the ratio of total to HDL-cholesterol [8, 9], all key risk factors of CVD, although it has been proposed that these changes are small and are not clinically significant [10].

Evidence for the association between egg consumption and glycaemic control is limited. Findings from a randomized controlled trial (RCT) of 140 overweight or obese subjects with prediabetes or T2D showed that high-egg consumption (2 eggs/day for 6 days/week) did not adversely effect lipid profiles of those with T2D [11]. Furthermore, Fuller et al. [11] reported no effect of egg intake on glycaemic control in a 3-month RCT in subjects with T2D, whereas an inverse association between egg intake and fasting plasma glucose was reported in a prospective cohort study [12]. Moreover, evidence from previous meta-analyses in relation to egg consumption and CVD risk showed inconsistent results. Some studies [13, 14] have shown that consumption of up to one egg per day is not associated with increased risk of CVD in the general population, which is in contrast to a recent meta-analysis which reported up to one egg per day was associated with reduced risk of stroke [15]. Furthermore, inconsistent associations between egg intake and CVD in diabetic patients were observed. Shin et al. [14] concluded that consumption of up to one egg per day was associated with an increased risk of CVD in those suffering from T2D, whereas Rong et al. [13] found intake of up to one egg per day was associated with an increased risk of CHD in diabetic patients, but may have contributed to a reduced risk of hemorrhagic stroke in this sub-group.

Due to the inconsistent reported relationship between egg consumption and cardio-metabolic disease incidence and risk we investigated the association between egg consumption and incidence of CVD (primary outcome) and all-cause mortality and incidence of T2D (secondary outcomes) in middle-aged men from the UK Caerphilly Prospective Cohort Study (CAPS). Furthermore, due to the different pathophysiologies of diseases classed as CVD, including myocardial infarction (MI), stroke and heart failure [16], the relationship between these diseases and egg consumption were further investigated separately. Furthermore, the cross-sectional relationships between egg intake and a number of cardiometabolic risk markers were investigated using data from the CAPS and the UK National Diet and Nutrition Survey (NDNS) rolling programme (years 1–4, secondary outcomes) cross-sectionally. Our hypothesis was that a higher egg intake was unlikely to increase the risk of CVD (including MI, stroke, heart failure and cardio-metabolic risk markers), T2D or all-cause mortality, but may have detrimental effects in those suffering from T2D.

## Subjects and methods

### Caerphilly prospective cohort study

#### Study population of caerphilly prospective cohort study

**Fig. 1:**
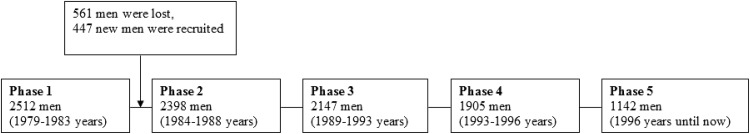
Timeline of the caerphilly prospective cohort study

The CAPS was set up between 1979 and 1983 and was designed to investigate CVD risk factors in men, there was follow-up of the men who were re-examined at 5-year intervals (Fig. 1). Initially, 2512 men aged 45–59-years-old living in Caerphilly, Wales, United Kingdom and the adjacent area were recruited onto the study [17]. However, 561 men decided to leave the study after Phase 1 (1979–1983) and an additional group of 447 men were recruited for replacement, giving a new total of 2398 men at Phase 2 (1984–1988). At 5 years later, a total of 2147 men revisited the clinical centre for the Phase 3 examination (1989–1993). The current study has not included data from Phase 1, as there were an inconsistent population and number of subjects between Phase 1 and Phase 2.

#### Dietary assessment

Diet was assessed at both Phase 2 and Phase 3 with the use of a semi-quantitative food-frequency questionnaire (FFQ). Participants were asked to report the number of eggs consumed on a weekly basis, with one unit of consumption equivalent to one egg, however, this excluded eggs present in composite dishes. This FFQ was previously validated using a 7-day weighed diet diary in a representative sub-group of 665 men, representing 30% of Phase 1 participants [18]. To present the best estimation of egg consumption, the mean egg intake of Phase 2 and Phase 3 was used for the longitudinal analysis, which also allowed estimation of the effect of the cumulative long-term diet. Subjects with pre-existing stroke (*n* = 60), MI (*n* = 98), and those with missing data (including subjects who dropped out during the follow-up period) or confounding factors (*n* = 208) were excluded from the longitudinal CVD analysis, which left a total of 1781 men. Subjects with pre-existing stroke, MI were also excluded from cross-sectional analyses at Phase 2 or Phase 3. Furthermore, subjects with pre-existing T2D (*n* = 94) were excluded from the longitudinal T2D analysis, which left a total 1687 men. The mean egg intakes at Phase 2 and Phase 3 were calculated for all of the subjects who reported egg consumption in both phases. Egg consumption (*n* = number/week) was divided into five groups for the analysis: 0 ≤ *n* ≤ 1, 1 < *n* ≤ 2, 2 < *n* ≤ 3, 3 < *n* < 5, *n* ≥ 5.

#### Cardiovascular events, diabetes and all-cause mortality

The incidence of T2D was self-reported from questionnaires in the CAPS. Identification of vascular disease events and deaths by cause has been described elsewhere [19–21]. In brief, subjects were seen in a central clinic, symptoms and illnesses suggestive of a stroke or heart attack were confirmed by the use of the London School of Hygiene chest pain questionnaire and the Oxford Stroke Questionnaire; subjects also had an electrocardiogram measurement during the visit. Appropriate searches of hospital and general practitioner databases were made to extract relevant clinical information. Vascular events (International Classification of Diseases (ICD) 121-5, 10th revision) of fatal ischaemic heart disease and non-fatal MI and ischaemic stroke (ICD 163-4) were diagnosed by two independent expert clinicians and an epidemiologist using all available clinical evidence, including computed tomography, radiological and pathological information. Furthermore, the records of all men in the National Health Service Central Registry were flagged so that notification of death certificate was received automatically, and cause of death was defined by ICD-9 Revision.

The aim of the present study was to investigate the relationship between egg intake and CVD or mortality in the total population, as well as in a sub-group suffering from T2D and/or impaired glucose tolerance (IGT). After removal of 16 subjects with T2D who had missing dietary or confounding factor data from baseline, 94 pre-existing T2D subjects remained for inclusion in the analysis. To have sufficient numbers for the statistical analysis, we combined the men with IGT (*n* = 319), defined by the WHO as fasting plasma glucose of 6.1 mmol/L or higher [22]. Subjects (*n* = 73) who met the inclusion criteria for both T2D and IGT were counted once in the analysis, thus, T2D and/or IGT sub-group included 340 subjects which were included in the longitudinal analysis of the associations between egg consumption with CVD events or all-cause mortality.

#### Other measurements

Systolic blood pressure (SBP), diastolic blood pressure (DBP), plasma glucose, insulin, triacylglycerol (TAG), total/HDL-cholesterol, fibrinogen, homocysteine and C-reactive protein (CRP) were measured in fasting plasma or serum samples at Phase 2. LDL-cholesterol was calculated using TAG, total cholesterol and HDL-cholesterol by the Friedewald formula [23]. Pulse pressure was calculated by subtracting DBP from SBP. However, only SBP, DPB, glucose, TAG, total cholesterol, fibrinogen were measured at Phase 3 in CAPS.

### National diet and nutrition survey

Data files from the National Diet and Nutrition Survey (NDNS) [24] years 1–4 of the rolling programme (2008–2009 to 2011–2012) were obtained from the UK Data Archive (http://www.data-archive.ac.uk). The data from 754 adults (males *n* = 322 and females *n* = 432) aged 19–64 years were used to determine association between egg intake (g/day) and fasting blood glucose, glycated haemoglobin (HbA1c) and other biochemical measures of cardio-metabolic health, including total-, HDL-, and LDL- cholesterol, the total-/HDL- cholesterol ratio, TAG, SBP, DBP, pulse pressure and CRP. Food intake was collected using 4-day food dairy. Participants with a previous history of stroke (*n* = 1), heart attack or angina (*n* = 6) were excluded from the analysis. In addition, associations between egg consumption and metabolic markers were examined in a T2D and/or IGT sub-group, which included subjects with T2D (*n* = 14) or IGT (fasting plasma glucose ≥ 6.1 mmol/L, *n* = 56), subjects (*n* = 11) who met inclusion criteria for both T2D and IGT groups were only counted once. Thus, the total number in the T2D and/or IGT sub-group was 59 subjects. The egg food group included whole eggs and dishes such as omelettes and scrambled eggs, which was analysed as continuous variable (with increments of 100 g/day). Composite dishes such as egg fried rice and quiches were removed from the food group to fit with the analysis conducted on the CAPS.

### Statistical analysis

#### Longitudinal investigation in CAPS

All data analysis was conducted using STATA (version 13.0; STATA Corporation, 2014) and a 2-sided *p* < 0.05 was considered statistically significant. In the longitudinal analysis of CAPS, Cox proportional hazard models were used to calculate non-adjusted and multivariate adjusted hazard ratios (HR) by comparing the time until onset of disease or mortality in subjects in the highest intake categories of egg consumption with that in the lowest egg group as the reference group. The survival time for each endpoint (MI, stroke, heart failure, T2D or mortality) was the first occurrence of an endpoint, death, or the date of receipt of the last follow-up questionnaire. If subjects had a non-fatal event these data were used rather than the later date of death.

The first multivariate model controlled for a number of confounding factors in CAPS. These included the covariates age (years), body mass index (weight (kg)/height (m^2^)), energy intake (kJ/day), alcohol consumption (as ethanol, ml/week), smoking (never-smoked, ex-smoked, current smoked), energy expenditure (kJ/day), social class (manual worker; non-manual worker), family history of MI (yes or no) and T2D (yes or no). The second multivariate model also controlled for sugar intake (< 50, 50–100, > 100 g/day), fruit consumption (< 7, 8–14, 15–21, or > 21 times/week), red meat consumption (< 7, 8–14, 15–21, or > 21 times/week) and fibre intake (cereal and vegetable sources) (< 10, 10–20, or > 20 g/day). The possibility of an interaction between egg consumption and subgroup of T2D and/or IGT with respect to any of the outcomes was investigated by an analysis including an interaction term in the Cox proportional hazard models.

#### Cross-sectional analysis in CAPS and NDNS

In secondary analyses, we examined the association between egg consumption and metabolic markers in CAPs and NDNS. For cross-sectional analysis of CAPs, the association of egg consumption with a range of metabolic markers was examined at Phase 2. In the sensitivity analyses of cross-sectional analysis, the associations of egg consumption with metabolic markers were also evaluated at Phase 3 to test the consistency of the findings. Trends associated with increasing egg consumption were investigated using linear regression for the continuous variables and logistic regression for categorical variables. The same confounding factors used for the multivariate models of the longitudinal analysis in CAPS were applied in the cross-sectional analysis.

For cross-sectional analysis of the NDNS, the confounding factors used were age (years), gender (men or women), energy intake (kJ/day), alcohol consumption (as ethanol, g/day), T2D (yes or no) and smoking habit (smokers or non-smokers). General linear regression was used for the analysis. Original data were transformed to natural logarithms if required.

## Results

### CAPS: baseline characteristics according to egg consumption

The mean egg intake of 1781 subjects was 2.9 (SD = 2.1) eggs per week, and ranged from 0 to 34 eggs per week. Among participants, 14.4% subjects consumed 5 eggs or more per week. The baseline characteristics of the subjects from the CAPS are shown in Table 1. The men in the highest quantiles of egg consumption were significantly more likely to be manual workers, smokers, consume more alcohol, have a higher energy intake and expenditure and a higher BMI. They also had a lower incidence of family MI history. After controlling for energy intake from foods, the men with the highest egg consumption had a significantly higher intake of total fat, saturated fat and sugar, but lower cereal or vegetable fibre, carbohydrate intakes, red meat and fruit intake.

**Table 1:** Baseline characteristics of participants from the caerphilly prospective cohort study according to egg consumption

Characteristics	Egg consumption (*n*, eggs/wk)					*P* trend^a^
	(0 ≤ n ≤ 1)	(1 < n ≤ 2)	(2 < n ≤ 3)	(3 < n < 5)	(n ≥ 5)	
Total subjects, *n*	274	464	469	318	256	
Age, years	61.5 ± 4.6	61.9 ± 4.3	61.7 ± 4.5	61.7 ± 4.5	61.6 ± 4.4	0.93
BMI, kg/m	26.5 ± 3.5	26.5 ± 3.4	26.7 ± 3.7	26.9 ± 4.0	27.1 ± 4.2	0.03
Energy expenditure, kJ/day	1440 ± 1557	1421 ± 1426	1551 ± 1651	1659 ± 1618	1850 ± 2042	0.001
Manual workers, %	53.3	59.7	63.5	70.8	80.1	< 0.001
Family history of MI, %	43.2	38.6	38.4	30.4	36.7	0.02
History of hypertension, %	28.8	26.9	29.2	31.1	29.7	0.40
History of diabetes, %	3.3	1.7	0.9	5.3	3.9	0.07
Current smokers, %	22.6	26.9	36.2	43.4	48.0	< 0.001
Total energy intake, kJ/day	7449 ± 1890	8074 ± 1873	8547 ± 2051	8988 ± 2132	9821 ± 2578	< 0.001
Fat, % of total energy	33.9	35.1	35.8	36.5	37.1	< 0.001
Saturated fat, % of total energy	14.6	15.0	15.5	15.9	16.1	< 0.001
Protein, % of total energy	14.7	14.3	14.3	14.2	14.5	0.19
Carbohydrates, % of total energy	49.3	48.3	47.6	47.1	45.9	< 0.001
Fibre (vegetable sources)^b^, g/day	11.4 ± 0.5	10.9 ± 0.5	11.1 ± 0.6	11.2 ± 0.6	11.3 ± 0.7	0.02
Fibre (cereal sources)^b^, g/day	10.8 ± 1.2	10.3 ± 1.1	10.0 ± 1.3	9.3 ± 1.3	9.2 ± 1.6	< 0.001
Sugar^b^, g/day	76.8 ± 24.1	82.2 ± 23.9	87.3 ± 26.1	93.6 ± 27.2	104.3 ± 32.9	< 0.001
Fruit^b^, number/week	9.3 ± 0.5	8.3 ± 0.5	7.9 ± 0.6	7.4 ± 0.6	7.7 ± 0.7	< 0.001
Vegetable^b^, times/week	10.7 ± 4.7	10.0 ± 4.6	10.5 ± 4.7	10.6 ± 4.8	10.3 ± 4.8	0.99
Red meat^b^, times/week	17.4 ± 0.9	14.9 ± 0.9	14.8 ± 1.0	14.3 ± 1.1	14.8 ± 1.3	< 0.001
Ethanol intake, ml/week	15.6 ± 21.5	16.2 ± 20.3	17.8 ± 22.5	17.3 ± 19.7	20.5 ± 22.8	0.010

All values are mean ± SD

^a^ *P*-trend was assessed by linear regression (continuous variables) or by logistic regression (categorical variables)

^b^Energy-adjusted values

### CAPS: egg consumption and CVD events, all-cause mortality and diabetes in longitudinal investigation

During the mean follow-up of 22.8 years, incident cases of total CVD event (*n* = 715) and all-cause mortality (*n* = 1028) were reported in the subjects initially free from CVD events (Table 2). When different types of CVD event were investigated separately, egg consumption was not associated with incidence of MI (*n* = 477) or heart failure (*n* = 201). However, a significant trend of higher risk of stroke with increasing egg intake (adjusted model *P* = 0.04) was observed, with HR of 1.60 (95% CI: 1.00, 2.57) for the highest (≥ 5 eggs/week) vs lowest (0 ≤ eggs/week ≤ 1) quantile of egg consumption.

**Table 2:** Longitudinal study of incidence of stroke, myocardial infarction, heart failure and all-cause mortality according to egg consumption of all subjects from the caerphilly prospective cohort study

Characteristics	Egg consumption (*n*, eggs/week)					*P*-trend
	(0 ≤ *n* ≤ 1)	(1 < *n* ≤ 2)	(2 < *n* ≤ 3)	(3 < *n* < 5)	(*n* ≥ 5)	
Total subjects, *n*	274	464	469	318	256	
Total CVD events						
No. of events	103	166	195	129	122	
HR (non-adjust)	1	0.95 (0.74, 1.21)	1.12 (0.88, 1.42)	1.08 (0.83, 1.40)	1.37 (1.05, 1.78)	0.007
HR (adjusted Model 1)^a^	1	0.97 (0.75, 1.24)	1.11 (0.87, 1.42)	1.02 (0.78, 1.34)	1.26 (0.95, 1.67)	0.09
HR (adjusted Model 2)^b^	1	0.98 (0.76, 1.26)	1.14 (0.89, 1.46)	1.01 (0.77, 1.33)	1.25 (0.94, 1.66)	0.12
Stroke						
No. of events	33	57	57	48	53	
HR (non-adjust)	1	1.01 (0.66, 1.56)	1.01 (0.66, 1.55)	1.27 (0.82, 1.98)	1.82 (1.18, 2.80)	0.002
HR (adjusted Model 1)^a^	1	0.99 (0.65, 1.53)	0.97 (0.63, 1.50)	1.14 (0.72, 1.81)	1.58 (1.00, 2.52)	0.03
HR (adjusted Model 2)^b^	1	1.01 (0.65, 1.56)	1.00 (0.64, 1.55)	1.15 (0.72, 1.84)	1.60 (1.00, 2.57)	0.04
Myocardial infarction						
No. of events	73	117	137	86	64	
HR (non-adjust)	1	0.94 (0.70, 1.26)	1.11 (0.83, 1.47)	1.00 (0.73, 1.37)	0.94 (0.67, 1.31)	0.98
HR (adjusted Model 1)	1	0.96 (0.71, 1.29)	1.10 (0.83, 1.48)	0.99 (0.71, 1.37)	0.90 (0.63, 1.29)	0.75
HR (adjusted Model 2)	1	0.97 (0.72, 1.31)	1.14 (0.85, 1.52)	1.01 (0.72, 1.40)	0.91 (0.64, 1.31)	0.80
Heart failure						
No. of events	29	33	63	44	32	
HR (non-adjust)	1	0.66 (0.40, 1.09)	1.29 (0.83, 2.00)	1.33 (0.83, 2.13)	1.20 (0.72, 1.98)	0.03
HR (adjusted Model 1)	1	0.64 (0.39, 1.06)	1.17 (0.74, 1.83)	1.10 (0.67, 1.79)	0.89 (0.52, 1.52)	0.46
HR (adjusted Model 2)	1	0.65 (0.39, 1.08)	1.19 (0.76, 1.88)	1.09 (0.66, 1.81)	0.89 (0.51, 1.53)	0.49
All-cause mortality						
No. of events	135	249	293	187	164	
HR (non-adjust)	1	1.13 (0.92, 1.40)	1.35 (1.10, 1.66)	1.25 (1.00, 1.56)	1.44 (1.14, 1.80)	0.001
HR (adjusted Model 1)	1	1.08 (0.87, 1.33)	1.21 (0.98, 1.49)	1.03 (0.82, 1.30)	1.11 (0.87, 1.42)	0.58
HR (adjusted Model 2)	1	1.08 (0.87, 1.34)	1.20 (0.98, 1.49)	1.02 (0.81, 1.29)	1.08 (0.84, 1.38)	0.80

^a^Values are hazard ratios (95% CIs) derived by Cox proportional hazards regression models adjusted for age (continuous), BMI (continuous), total energy intake (continuous), alcohol consumption (quartiles), smoking (never, past or current), energy expenditure (quartiles), social class (manual or non-manual), family history of myocardial infarction (yes or no), diabetes mellitus (yes or no)

^b^Adjusted as model 1 plus sugar intake (< 50, 50–100, > 100 g/day), fruit consumption (< 7, 8–14, 15–21, or > 21 times/week), red meat consumption (< 7, 8–14, 15–21, or > 21 times/week) and fibre (cereal and vegetable sources) (< 10, 10–20, or > 20 g/day)

In stratified analyses, the prevalence of T2D and/or IGT did not influence the association between egg consumption and total CVD, MI, heart failure, or all-cause mortality (data not shown). When the subjects with T2D and/or IGT were removed from the analysis of stroke, there was no significant increase in risk of stroke across increasing quantiles of egg consumption (Table 3). However, when this analysis was performed on the T2D and/or IGT sub-group, a significant trend for increased risk of stroke (adjusted model *P* = 0.01) with increasing egg consumption was identified, HR for incident stroke was 2.87 (95% CI 1.13, 7.27) in the highest vs lowest quantile of egg consumption (*P* for interaction between egg consumption and T2D and/or IGT = 0.09 in non-adjust model, 0.08 in adjusted Model 1 and 0.07 in adjusted Model 2).

**Table 3:** Longitudinal analysis of incident of stroke according to egg consumption in subjects with and without type 2 diabetes and/or impaired glucose tolerance from the Caerphilly Prospective Cohort study

	Egg consumption (n, eggs/week)					*P*-trend
	(0 ≤ *n* ≤ 1)	(1 < *n* ≤ 2)	(2 < *n* ≤ 3)	(3 < *n* < 5)	(*n* ≥ 5)	
Subjects without T2D and/or IGT^a^						
Total subjects, *n*	221	397	378	248	197	
No. of events	25	47	45	34	31	
HR (non-adjust)	1	1.05 (0.64, 1.70)	1.05 (0.65, 1.72)	1.23 (0.73, 2.05)	1.45 (0.85, 2.45)	0.12
HR (adjusted Model 1)^b^	1	1.01 (0.62, 1.66)	0.97 (0.59, 1.60)	1.14 (0.67, 1.95)	1.28 (0.73, 2.24)	0.33
HR (adjusted Model 2)^c^	1	1.05 (0.64, 1.71)	1.01 (0.61, 1.66)	1.17 (0.68, 2.02)	1.32 (0.75, 2.34)	0.29
Subjects with T2D and/or IGT						
Total Subjects, n	53	67	91	70	59	
No. of events	8	10	12	14	22	
HR (non-adjust)	1	0.96 (0.38, 2.44)	0.86 (0.35, 2.10)	1.35 (0.57, 3.23)	2.71 (1.21, 6.09)	0.003
HR (adjusted Model 1)	1	1.10 (0.42, 2.86)	1.02 (0.40, 2.62)	1.35 (0.53, 3.43)	2.83 (1.15, 6.96)	0.01
HR (adjusted Model 2)	1	1.09 (0.41, 2.88)	0.96 (0.37, 2.50)	1.39 (0.54, 3.56)	2.87 (1.13, 7.27)	0.01

^a^Impaired glucose tolerance, i.e., fasting glucose ≥ 6.1 mmol/L

^b^Values are hazard ratios (95% CIs) derived by Cox proportional hazards regression models adjusted for age (continuous), BMI (continuous), total energy intake (continuous), alcohol consumption (quartiles), smoking (never, past or current), energy expenditure (quartiles), social class (manual or non-manual), family history of myocardial infarction (yes or no)

^c^Adjusted as model 1 plus sugar intake (< 50, 50–100, > 100 g/day), fruit consumption (< 7, 8–14, 15–21, or > 21 times/week), red meat consumption (< 7, 8–14, 15–21, or > 21 times/week) and fibre (cereal and vegetable sources) (< 10, 10–20, or > 20 g/day)

During the follow-up, a total of 120 new T2D cases were diagnosed for subjects initially free from CVD and T2D events. There was no association between egg consumption and incident T2D using either un-adjusted or multiple adjusted model (Table 4).

**Table 4:** Longitudinal study of incidence of type 2 diabetes according to egg consumption from the caerphilly prospective cohort study

	Egg consumption (*n*, eggs/week)					*P*-trend
	(0 ≤ *n* ≤ 1)	(1 < *n* ≤ 2)	(2 < *n* ≤ 3)	(3 < *n* < 5)	(*n* ≥ 5)	
Total subjects, *n*	259	447	453	290	238	
No. of events	17	31	35	21	16	
HR (non-adjust)	1	1.17 (0.65, 2.12)	1.24 (0.69, 2.21)	1.20 (0.63, 2.27)	1.22 (0.61, 2.41)	0.59
HR (adjusted Model 1)^a^	1	1.08 (0.59, 1.97)	1.05 (0.57, 1.92)	1.25 (0.64, 2.44)	1.23 (0.60, 2.53)	0.48
HR (adjusted Model 2)^b^	1	1.05 (0.57, 1.93)	1.02 (0.55, 1.88)	1.24 (0.63, 2.45)	1.31 (0.63, 2.73)	0.39

^a^Values are hazard ratios (95% CIs) derived by Cox proportional hazards regression models adjusted for age (continuous), BMI (continuous), total energy intake (continuous), alcohol consumption (quartiles), smoking (never, past or current), energy expenditure (quartiles), social class (manual or non-manual), family history of myocardial infarction (yes or no)

^b^Adjusted as model 1 plus sugar intake (< 50, 50–100, > 100 g/d), fruit consumption (< 7, 8–14, 15–21, or > 21 times/week), red meat consumption (< 7, 8–14, 15–21, or > 21 times/week) and fibre (cereal and vegetable sources) (< 10, 10–20, or > 20 g/day)

### CAPS and NDNS: associations between egg consumption and cardio-metabolic risk factors: cross-sectional analysis

In Phase 2 of the CAPS, no associations were found between egg consumption and fasting plasma glucose concentration in the subjects free from CVD (Supplemental Table 1). However, when the analysis was repeated in a subgroup (*n* = 268) of participants with T2D and/or IGT, there was a significant positive association between egg consumption and fasting glucose concentration (Supplemental Table 2). In this subgroup analysis subjects consuming ≥ 5 eggs/week had 1.31 mmol/L higher fasting glucose compared with subjects consuming 1 eggs/wk or less. There were no significant associations between egg consumption and other biomarkers of CVD risk in the population as a whole (Supplemental Table 1) and in subgroup analysis (Supplemental Table 2).

In Phase 3 of the CAPS there was a significant positive association between egg consumption and fasting glucose concentration (Supplemental Table 3). When the subjects with T2D and/or IGT were removed from the analysis, there was no association between egg consumption and fasting glucose concentration across increasing quantile of egg consumption. However, when the analysis was repeated in the subgroup of T2D and/or IGT (*n* = 334) there was a significant positive association between egg consumption and fasting glucose (Supplemental Table 4). In this subgroup analysis subjects consuming ≥ 5 eggs per week had 0.72 mmol/L higher fasting glucose levels compared with subjects consuming ≤ 1 eggs/week.

Cross-sectional analysis in NDNS [24] showed egg consumption was positively associated with HbA1c concentrations (Supplemental Table 5). When the subjects with T2D and/or IGT were removed from the analysis, there remained a significant positive trend for increased egg consumption and HbA1c concentration (*P* = 0.02). In the T2D and/or IGT subgroup, egg consumption was significantly associated with elevated fasting glucose and HbA1c concentrations (*P* = 0.05; Supplemental Table 6). There were no significant associations between egg consumption and other markers of cardiovascular risk (Supplemental Table 5).

## Discussion

In the CAPS no overall associations between weekly egg consumption up to 5 eggs/week and risk of total CVD, MI, heart failure, all-cause mortality and T2D were observed after a mean follow-up of 22.8 years, but there was a significant positive trend in stroke risk across the quantiles of egg intake. Further sub-group analysis showed that the significant trend disappeared after the men with T2D and/or IGT were removed. However the association of egg intake and risk of stroke in the sub-group of T2D and/or IGT only, showed significant positive associations. Secondary analyses in both CAPS and NDNS showed increased fasting glucose with higher egg intake in the sub-group of T2D and/or IGT. In addition, results of cross-sectional analyses of NDNS showed higher egg intake was associated with higher HbA1c in the general healthy population across tertiles of egg intake.

These results are consistent with the previous meta-analyses of prospective studies [13, 14], which showed no association between egg consumption and CVD events in the general population. Very few studies have reported a positive association between egg intake and CVD risk. Nevertheless, data from Physicians’ Health Study [25] indicated the HR of heart failure was 1.33 (95% CI 1.04, 1.70) with egg consumption ≥ 7 per week compared with that of < 1 per week over 20 years of follow-up for physicians free of previous MI. In addition, in another analysis of the same cohort there was no association between egg intake and MI and stroke, but there was a significant positive association with all-cause mortality [26]. The difference in the observed association of egg consumption and heart failure or all-cause mortality between the current study and that by Djousse et al. [25, 26] may be due to differences in the characteristics of the investigated subjects. Physicians were included in the study of Djousse et al. [25, 26], whereas 64% of the men in the current study were manual workers. An earlier British study in 1997 reported a 2.68 times increased risk of ischaemic heart disease deaths in 10,802 subjects reporting higher egg intake (> 6/week) compared with lower egg consumption (< 1 egg/week) after 13.3 years follow-up [27]. In our study we were not able to conduct a similar analysis, as the data for mortality resulting from different categories of heart disease were not available.

Our finding of no association between egg intake and risk of stroke in generally healthy men is consistent with previous studies [28–30]. However, in our sub-group analysis of 340 men with T2D and/or IGT, we found a significant positive association between egg intake and the risk of stroke. A few epidemiological studies [13, 14] have investigated the association of egg consumption and CVD in T2D subjects, but none have included participants with IGT. A recent review has summarised the conflicting evidence and concluded that a diet including more eggs than is recommended (at least in some countries) may be used safely for all of the population which includes those with T2D [31]. Our study is not totally supportive of this statement and to the best of our knowledge; we are the first to show a positive association between egg consumption and stroke in subjects with T2D and/or IGT, which needs confirmation in further studies.

In our secondary cross-sectional analysis of CAPS and NDNS, a positive association between egg intake and blood glucose was observed in the T2D and/or IGT subgroup. This may indicate that higher egg intake had a detrimental effect on the glucose metabolism in subjects with T2D and/or IGT, although this needs confirmation in randomized controlled dietary intervention trials. An earlier study also showed that higher egg consumption was associated with elevated fasting glucose in 394 middle-aged healthy men [7]. This is also supported by another cross-sectional analysis [32] that observed significant positive relationships between egg consumption and fasting glucose, insulin or insulin resistance, although the difference was very small. However, neither of these studies investigated the association in sub-groups with T2D and/or IGT [7, 32].

The concept of eggs as a cholesterol rich food, which may increase LDL-cholesterol and risk of heart disease, has been recognised for a long time. However, in our analysis, we found no association between egg intake and blood cholesterol concentrations, in agreement with Gray and Griffin [10] who concluded that the effect of dietary cholesterol on LDL-cholesterol was negligible compared with the effect of dietary saturated fatty acids. In contrast to our findings, a study in a Finnish population showed a reduction in fasting plasma glucose in the highest egg consumption quartile (> 45 g/day) at baseline and after a 4-year follow-up in 2312 men [12]. This significant association at baseline only appeared when dietary cholesterol intake was included as a covariate. However, controlling for dietary cholesterol may lead to bias as this component of eggs could be responsible for the observed effects of eggs on plasma glucose [33].

Lastly, this is the first study to show a strong positive association between higher egg intake and elevated HbA1c concentration in a population without CVD or known T2D by using NDNS. Higher HbA1c is regarded as an important marker for pre-diagnosed T2D and CVD [34, 35]. There was no evidence in CAPS of any association between egg consumption and the development of T2D. However in a larger study, Djousse and colleagues [36] showed a significant positive association between egg consumption (≥ 7 weekly) and risk of T2D in two large prospective cohort studies of men (*n* = 20,303) and women (*n* = 36,295) with 1921 and 2112 cases of T2D after a follow-up period of 20.0 years for men and 11.7 years for women, respectively. One possible explanation for the non-significant finding in CAPS is the numbers of self-reported T2D cases were relatively small and were not validated by clinical diagnosis. Nevertheless, our study agrees with the recent meta-analysis [37] which identified 12 prospective cohort studies and showed infrequent egg consumption was not associated with risk of T2D.

The potential mechanism by which eggs could increase fasting plasma glucose and ischaemic stroke in those with T2D and/or IGT is unclear. Findings from a 3-month randomized controlled study showed that there was no negative effect of higher egg consumption (> 12 eggs/week) on blood lipid profile compared with low egg consumption (< 2 eggs/week) in overweight or obese subjects diagnosed with diabetes or prediabetes [11]. However, in that study the authors controlled for diabetes or prediabetes drug use which may have masked the effect of egg consumption.

The major strength of the CAPS study is that it has a follow-up of over 20 years, which is one of the longest UK prospective cohort studies providing new evidence on the relationship between dietary factors and CVD events. Furthermore, to prevent chance findings, the cross-sectional analyses were repeated in two phases (Phase 2 and 3) of the CAPS (5-year interval). However, egg consumption was only recorded as the weekly egg intake and did not account for eggs consumed from composite dishes or purchased foods. Thus, egg intakes may have been underestimated or misclassified, which may have affected the observed associations. In addition, cooking methods and information on how eggs were consumed were not recorded, which may have had effects on health outcomes [38]. In terms of the metabolic markers available in CAPS, insulin was only measured in a small proportion of subjects and was not measured in Phase 3, thus insulin resistance could not be estimated.

In conclusion, our study showed no evidence for adverse effects of infrequent egg intake (3 eggs per week) on the risk of CVD, T2D and all-cause mortality in healthy men. However, a detrimental association of infrequent egg intake (3 eggs per week) on fasting glucose and risk of ischaemic stroke in T2D and/or IGT subjects was observed and an adverse cross-sectional association of egg consumption on HbA1C in a generally healthy population. However, due to the limited sample size and number of events and deaths, cautious interpretation of these results is recommended. In addition, residual confounding factors may have influenced the association between egg consumption and health outcomes [39]. As eggs are rarely consumed alone, further research should explore the dietary pattern of high and low egg consumers, and large prospective cohort studies and RCTs are required to verify these findings in the future.

## Electronic supplementary material

Below is the link to the electronic supplementary material.

Supplementary material 1 (DOCX 76 KB)

## Abbreviations

CVD: Cardiovascular disease
MI: Myocardial infarction
T2D: Type 2 diabetes
IGT: Impaired glucose tolerance
CAPS: Caerphilly prospective cohort study
NDNS: National diet and nutrition survey
FFQ: Food-frequency questionnaire
RCT: Randomized controlled trials
SBP: Systolic blood pressure
DBP: Diastolic blood pressure
TAG: Triacylglycerol
CRP: C-reactive protein
HbA1c: Glycated haemoglobin
ICD: International Classification of Diseases
HR: Hazard ratio
