# Supplementary material for: Association between egg consumption and cardiovascular disease events, diabetes and all-cause mortality
Source: Eur J Nutr. 2017 Nov 2;57(8):2943–52. doi: 10.1007/s00394-017-1566-0 (PMC6267402; doi:10.1007/s00394-017-1566-0)
Supplement: Supplementary file 1 — Supplementary material 1 (DOCX 76 KB) [file 394_2017_1566_MOESM1_ESM.docx]

Electronic Supplementary Material for European Journal of Nutrition

**Title: Association between egg consumption and cardiovascular disease events, diabetes and all-cause mortality**

Jing Guo, Ditte A Hobbs, John R Cockcroft, Peter C Elwood, Janet E Pickering, Julie A Lovegrove and David I Givens

^1^ Institute for Food, Nutrition and Health (JG, JEP, JAL, DIG), the Hugh Sinclair Unit of Human Nutrition (DAH, JAL) and the Institute for Cardiovascular and Metabolic Research (JG, DAH, DIG, JAL) University of Reading, Reading, RG6 6AR, United Kingdom; Wales Heart Research Institute (JRC), and Department of Primary Care and Public Health (PCE), Cardiff University, United Kingdom.

^2^ Address correspondence to Julie A. Lovegrove, Hugh Sinclair Unit of Human Nutrition and Institute for Cardiovascular and Metabolic Research, University of Reading, University of Reading, Reading, RG6 6AR, United Kingdom. E-mail: j.a.lovegrove@reading.ac.uk. Telephone: +44(0)1183786418.

^3^ Supported by the Barham Benevolent Foundation.

**Supplemental Table 1** Cross-sectional (Phase 2) analysis of metabolic markers across egg consumption categories of all subjects in Caerphilly Prospective Cohort Study^1^

|  | Egg consumption (n, eggs/wk) | | | | |  |
| --- | --- | --- | --- | --- | --- | --- |
| Characteristics | (0≤ n ≤1) | (1< n ≤2) | (2< n ≤3) | (3< n <5) | (n ≥5) | *P*-trend |
| Glucose^4^ |  |  |  |  |  |  |
| Participants, n | 413 | 562 | 415 | 347 | 421 |  |
| Mean, mmol/L | 5.25 (0.84) | 5.43 (1.43) | 5.36 (1.26) | 5.41 (1.24) | 5.49 (1.56) |  |
| Coef. (Std. Err.) non-adjust | Reference | 0.02 (0.00, 0.05) | 0.01 (-0.01, 0.04) | 0.02 (-0.00, 0.05) | 0.03 (0000, 0.05) | 0.05 |
| Coef. (Std. Err.) adjusted Model 1^2^ | Reference | 0.02 (-0.00, 0.04) | 0.01 (-0.01, 0.03) | 0.02 (-0.00, 0.04) | 0.01 (-0.01, 0.03) | 0.44 |
| Coef. (Std. Err.) adjusted Model 2^3^ | Reference | 0.02 (-0.00, 0.04) | 0.01 (-0.02, 0.03) | 0.01 (-0.01, 0.04) | 0.01 (-0.02, 0.03) | 0.70 |
| Insulin^4^ |  |  |  |  |  |  |
| Participants, n | 200 | 272 | 196 | 169 | 205 |  |
| Mean, mmol/L | 5.28 (22.19) | 3.74 (3.75) | 3.64 (4.21) | 3.17 (2.32) | 3.43 (2.86) |  |
| Coef. (Std. Err.) non-adjust | Reference | 0.00 (-0.13, 0.13) | -0.01 (-0.15, 0.13) | -0.09 (-0.24, 0.06) | -0.06 (-0.20, 0.08) | 0.20 |
| Coef. (Std. Err.) adjusted Model 1 | Reference | -0.01 (-0.14, 0.12) | -0.03 (-0.17, 0.11) | -0.13 (-0.28, 0.02) | -0.09 (-0.24, 0.07) | 0.10 |
| Coef. (Std. Err.) adjusted Model 2 | Reference | -0.02 (-0.15, 0.11) | -0.03 (-0.17, 0.11) | -0.13 (-0.28, 0.03) | -0.08 (-0.23, 0.08) | 0.15 |
| Total cholesterol |  |  |  |  |  |  |
| Participants, n | 409 | 564 | 414 | 348 | 424 |  |
| Mean, mmol/L | 5.64 (0.99) | 5.56 (0.99) | 5.62 (1.05) | 5.64 (1.02) | 5.68 (1.00) |  |
| Coef. (Std. Err.) non-adjust | Reference | -0.08 (-0.21, 0.05) | -0.02 (-0.16, 0.12) | -0.00 (-0.15, 0.14) | 0.04 (-0.10, 0.18) | 0.26 |
| Coef. (Std. Err.) adjusted Model 1 | Reference | -0.09 (-0.22, 0.04) | -0.01 (-0.15, 0.13) | -0.00 (-0.15, 0.15) | 0.05 (-0.09, 0.20) | 0.18 |
| Coef. (Std. Err.) adjusted Model 2 | Reference | -0.09 (-0.22, 0.04) | -0.03 (-0.17, 0.11) | -0.02 (-0.17, 0.13) | 0.03 (-0.11, 0.18) | 0.35 |
| HDL-cholesterol, |  |  |  |  |  |  |
| Participants, n | 409 | 564 | 414 | 348 | 424 |  |
| Mean, mmol/L | 1.03 (0.25) | 1.01 (0.24) | 1.03 (0.24) | 1.02 (0.25) | 1.04 (0.27) |  |
| Coef. (Std. Err.) non-adjust | Reference | -0.02 (-0.06, 0.01) | 0.00 (-0.03, 0.03) | -0.01 (-0.04, 0.03) | 0.01 (-0.03, 0.04) | 0.36 |
| Coef. (Std. Err.) adjusted Model 1 | Reference | -0.02 (-0.05, 0.01) | 0.01 (-0.03, 0.04) | 0.01 (-0.02, 0.05) | 0.02 (-0.01, 0.06) | 0.05 |
| Coef. (Std. Err.) adjusted Model 2 | Reference | -0.02 (-0.05, 0.01) | 0.00 (-0.03, 0.04) | 0.01 (-0.03, 0.04) | 0.02 (-0.02, 0.05) | 0.08 |
| LDL-cholesterol^4^ |  |  |  |  |  |  |
| Participants, n | 409 | 564 | 414 | 348 | 424 |  |
| Mean, mmol/L | 4.23 (0.94) | 4.15 (0.90) | 4.22 (0.96) | 4.21 (0.96) | 4.25 (0.92) |  |
| Coef. (Std. Err.) non-adjust | Reference | -0.08 (-0.20, 0.03) | -0.01 (-0.14, 0.12) | -0.02 (-0.16, 0.11) | 0.02 (-0.11, 0.14) | 0.43 |
| Coef. (Std. Err.) adjusted Model 1 | Reference | -0.09 (-0.21, 0.02) | -0.00 (-0.13, 0.12) | -0.03 (-0.16, 0.11) | 0.04 (-0.10, 0.17) | 0.26 |
| Coef. (Std. Err.) adjusted Model 2 | Reference | -0.09 (-0.21, 0.03) | -0.02 (-0.14, 0.11) | -0.04 (-0.17, 0.10) | 0.03 (-0.11, 0.16) | 0.40 |
| Triglycerides^4^ |  |  |  |  |  |  |
| Participants, n | 409 | 564 | 414 | 348 | 424 |  |
| Mean, mmol/L | 1.89 (1.06) | 2.04 (1.80) | 1.86 (1.11) | 2.04 (1.57) | 1.98 (1.29) |  |
| Coef. (Std. Err.) non-adjust | Reference | 0.03 (-0.03, 0.10) | -0.02 (-0.09, 0.05) | 0.04 (-0.04, 0.11) | 0.03 (-0.04, 0.10) | 0.55 |
| Coef. (Std. Err.) adjusted Model 1 | Reference | 0.01 (-0.05, 0.08) | -0.03 (-0.10, 0.03) | -0.01 (-0.08, 0.07) | -0.03 (-0.10, 0.04) | 0.30 |
| Coef. (Std. Err.) adjusted Model 2 | Reference | 0.01 (-0.05, 0.07) | -0.04 (-0.11, 0.03) | -0.01 (-0.09,0.06) | -0.04 (-0.11, 0.03) | 0.18 |
| Fibrinogen^4^ |  |  |  |  |  |  |
| Participants, n | 252 | 340 | 280 | 236 | 253 |  |
| Mean, g/L | 3.03 (0.78) | 3.09 (0.85) | 3.07 (0.85) | 3.09 (0.89) | 3.14 (0.83) |  |
| Coef. (Std. Err.) non-adjust | Reference | 0.02 (-0.02, 0.06) | 0.01 (-0.03, 0.05) | 0.01 (-0.03, 0.06) | 0.03 (-0.01, 0.08) | 0.23 |
| Coef. (Std. Err.) adjusted Model 1 | Reference | 0.00 (-0.04, 0.04) | -0.00 (-0.04, 0.04) | -0.01 (-0.06, 0.03) | 0.02 (-0.02, 0.07) | 0.55 |
| Coef. (Std. Err.) adjusted Model 2 | Reference | 0.01 (-0.03, 0.04) | -0.00 (-0.06, 0.03) | -0.01 (-0.06, 0.03) | 0.02 (-0.03, 0.06) | 0.77 |
| Homocysteine |  |  |  |  |  |  |
| Participants, n | 412 | 560 | 408 | 346 | 424 |  |
| Mean, mmol/L | 12.65 (4.91) | 12.28 (5.07) | 12.59 (4.77) | 11.91 (4.81) | 12.37 (5.52) |  |
| Coef. (Std. Err.) non-adjust | Reference | -0.36 (-1.00, 0.28) | -0.05 (-0.74, 0.64) | -0.73 (-1.45, -0.01) | -0.28 (-0.96, 0.40) | 0.30 |
| Coef. (Std. Err.) adjusted Model 1 | Reference | -0.37 (-1.01, 0.27) | -0.12 (-0.80, 0.57) | -0.74 (-1.47, -0.02) | -0.35 (-1.07, 0.36) | 0.24 |
| Coef. (Std. Err.) adjusted Model 2 | Reference | -0.41 (-1.04, 0.22) | -0.21 (-1.55, 0.47) | -0.83 (-1.55, -0.10) | -0.61 (-1.33, 0.11) | 0.06 |
| C-reactive protein^4^ |  |  |  |  |  |  |
| Participants, n | 280 | 395 | 285 | 242 | 288 |  |
| Mean, mg/L | 2.42 (2.78) | 3.29 (5.30) | 2.94 (4.91) | 2.96 (4.00) | 3.22 (4.35) |  |
| Coef. (Std. Err.) non-adjust | Reference | 0.18 (0.02, 0.33) | 0.10 (-0.07, 0.27) | 0.16 (-0.02, 0.34) | 0.17 (0.00, 0.34) | 0.13 |
| Coef. (Std. Err.) adjusted Model 1 | Reference | 0.10 (-0.05, 0.25) | 0.06 (-0.10, 0.22) | 0.03 (-0.14, 0.20) | 0.04 (-0.13, 0.21) | 0.98 |
| Coef. (Std. Err.) adjusted Model 2 | Reference | 0.0 (-0.05, 0.24) | 0.04 (-0.12, 0.20) | 0.02 (-0.15, 0.19) | -0.01 (-0.18, 0.16) | 0.54 |
| Systolic blood pressure^4^ |  |  |  |  |  |  |
| Participants, n | 427 | 577 | 427 | 356 | 433 |  |
| Mean, mmHg | 147.0 (22.1) | 146.5 (22.2) | 144.8 (23.4) | 148.1 (23.5) | 146.1 (22.6) |  |
| Coef. (Std. Err.) non-adjust | Reference | -0.00 (-0.02, 0.02) | -0.02 (-0.04, 0.00) | 0.01 (-0.01, 0.03) | -0.01 (-0.03, 0.01) | 0.85 |
| Coef. (Std. Err.) adjusted Model 1 | Reference | -0.01 (-0.03, 0.01) | -0.02 (-0.04, -0.00) | -0.00 (-0.02, 0.02) | -0.02 (-0.04, 0.00) | 0.19 |
| Coef. (Std. Err.) adjusted Model 2 | Reference | -0.01 (-0.03, 0.01) | -0.02 (-0.04, -0.00) | -0.00 (-0.02, 0.02) | -0.02 (-0.04, -0.00) | 0.14 |
| Diastolic blood pressure |  |  |  |  |  |  |
| Participants, n | 426 | 577 | 427 | 356 | 433 |  |
| Mean, mmHg | 84.7 (11.3) | 85.3 (11.7) | 83.9 (12.0) | 85.5 (12.4) | 85.0 (13.3) |  |
| Coef. (Std. Err.) non-adjust | Reference | 0.59 (-0.93, 2.11) | -0.75 (-2.38, 0.87) | 0.83 (-0.88, 2.53) | 0.30 (-1.33, 1.92) | 0.75 |
| Coef. (Std. Err.) adjusted Model 1 | Reference | 0.44 (-1.04, 1.93) | -0.80 (-2.39, 0.79) | 0.49 (-1.21, 2.19) | -0.09 (-1.77, 1.60) | 0.83 |
| Coef. (Std. Err.) adjusted Model 2 | Reference | 0.48 (-1.00, 1.97) | -0.77 (-2.37, 0.82) | 0.54 (-1.16, 2.25) | -0.34 (-2.03, 1.35) | 0.64 |

^1^ All values are mean ± SD.

^2^ *P*-trend was assessed by linear regression (continuous variables) or by logistic regression (categorical variables), adjusted for age (continuous), BMI (continuous), total energy intake (continuous), alcohol consumption (quartiles), smoking (never, past or current), energy expenditure (quartiles), social class (manual or non-manual), family history of myocardial infarction (yes or no), diabetes mellitus (yes or no).

^3^Adjusted as model 1 plus sugar intake (<50, 50-100, >100 g/d), fruit consumption (<7, 8-14, 15-21, or >21 times/wk), red meat consumption (<7, 8-14, 15-21, or >21 times/wk) and fibre (cereal and vegetable sources) (<10, 10-20, or >20 g/d).

^4^ Original data were transformed to natural logarithms for regression model.

**Supplemental Table 2** Cross-sectional (Phase 2) analysis of metabolic markers across egg consumption in subjects with type 2 diabetes and/or impaired glucose tolerance from the Caerphilly Prospective Cohort study^1^

|  | Egg consumption (n, eggs/wk) | | | | |  |
| --- | --- | --- | --- | --- | --- | --- |
| Characteristics | (0≤ n ≤1) | (1< n ≤2) | (2< n ≤3) | (3< n <5) | (n ≥5) | *P*-trend |
| Glucose^4^ |  |  |  |  |  |  |
| Participants, n | 42 | 72 | 46 | 46 | 62 |  |
| Fasting glucose, mmol/L | 6.89 (1.38) | 7.88 (2.83) | 7.73 (2.60) | 7.45 (2.37) | 8.20 (2.62) |  |
| Coef.± Std. Err. (non-adjust) | Reference | 0.10 (0.00, 0.21) | 0.09 (-0.02, 0.20) | 0.06 (-0.05, 0.20) | 0.15 (0.05, 0.26) | 0.04 |
| Coef ± Std. Err. (adjusted Model 1)^2^ | Reference | 0.12 (0.02, 0.22) | 0.11 (0.00, 0.23) | 0.07 (-0.04, 0.18) | 0.19 (0.08, 0.30) | 0.008 |
| Coef ± Std. Err. (adjusted Model 2)^3^ | Reference | 0.14 (0.03, 0.24) | 0.11 (-0.00, 0.22) | 0.05 (-0.06, 0.17) | 0.16 (0.05, 0.27) | 0.02 |
| Insulin^4^ |  |  |  |  |  |  |
| Participants, n | 22 | 39 | 21 | 21 | 28 |  |
| Mean, mmol/L | 3.78 (2.60) | 3.79 (3.49) | 3.60 (1.65) | 2.95 (1.54) | 3.06 (1.97) |  |
| Coef.± Std. Err. (non-adjust) | Reference | 0.02 (-0.37, 0.41) | 0.10 (-0.35, 0.54) | -0.16 (-0.35, 0.54) | -0.08 (-0.49, 0.34) | 0.46 |
| Coef ± Std. Err. (adjusted Model 1) | Reference | 0.05 (-0.37, 0.47) | 0.10(-0.36, 0.57) | -0.19 (-0.67, 0.29) | -0.15 (-0.60, 0.30) | 0.29 |
| Coef ± Std. Err. (adjusted Model 2) | Reference | 0.04 (-0.43, 0.52) | 0.07 (-0.47, 0.60) | -0.31 (-0.86, 0.25) | -0.21 (-0.70, 0.29) | 0.17 |
| Total cholesterol |  |  |  |  |  |  |
| Participants, n | 42 | 72 | 45 | 45 | 62 |  |
| Mean, mmol/L | 5.43 (0.75) | 5.69 (1.37) | 5.37 (0.98) | 5.61 (1.03) | 5.54 (1.15) |  |
| Coef.± Std. Err. (non-adjust) | Reference | 0.26 (-0.17, 0.69) | -0.06 (-0.53, 0.42) | 0.18 (-0.29, 0.66) | 0.11 (-0.33, 0.55) | 0.94 |
| Coef ± Std. Err. (adjusted Model 1) | Reference | 0.23 (-0.19, 0.65) | 0.00 (-0.46, 0.46) | 0.27 (-0.20, 0.74) | 0.19 (-0.25, 0.64) | 0.50 |
| Coef ± Std. Err. (adjusted Model 2) | Reference | 0.22 (-0.21, 0.66) | 0.02 (-0.46, 0.50) | 0.25 (-0.24, 0.74) | 0.15 (-0.31, 0.60) | 0.65 |
| HDL-cholesterol, |  |  |  |  |  |  |
| Participants, n | 42 | 72 | 45 | 45 | 62 |  |
| Mean, mmol/L | 1.05 (0.24) | 0.91 (0.23) | 0.95 (0.24) | 1.00 (0.26) | 0.99 (0.26) |  |
| Coef.± Std. Err. (non-adjust) | Reference | -0.15 (-0.24, -0.05) | -0.10 (-0.21, 0.00) | -0.05 (-0.16, 0.05) | -0.07 (-0.16, 0.03) | 0.82 |
| Coef ± Std. Err. (adjusted Model 1) | Reference | -0.18 (-0.27, -0.08) | -0.11 (-0.21, -0.01) | -0.03 (-0.13, 0.07) | -0.04 (-0.14, 0.06) | 0.24 |
| Coef ± Std. Err. (adjusted Model 2) | Reference | -0.18 (-0.27, -0.09) | -0.12 (-0.22, -0.12) | -0.04 (-0.14, 0.07) | -0.05 (-0.14, 0.05) | 0.33 |
| LDL-cholesterol^4^ |  |  |  |  |  |  |
| Participants, n | 42 | 72 | 45 | 45 | 62 |  |
| Mean, mmol/L | 4.00 (0.73) | 4.18 (1.10) | 3.95 (0.85) | 4.18 (1.01) | 1.08 (1.00) |  |
| Coef.± Std. Err. (non-adjust) | Reference | 0.18 (-0.19, 0.55) | -0.04 (-0.45, 0.37) | 0.18 (-0.23, 0.59) | 0.09 (-0.29, 0.47) | 0.83 |
| Coef ± Std. Err. (adjusted Model 1) | Reference | 0.18 (-0.18, 0.54) | 0.01 (-0.38, 0.41) | 0.28 (-0.13, 0.69) | 0.19 (-0.19, 0.57) | 0.34 |
| Coef ± Std. Err. (adjusted Model 2) | Reference | 0.17 (-0.21, 0.54) | 0.17 (-0.20, 0.54) | 0.05 (-0.36, 0.46) | 0.16 (-0.23, 0.55) | 0.39 |
| Triglycerides^4^ |  |  |  |  |  |  |
| Participants, n | 42 | 72 | 45 | 45 | 62 |  |
| Mean, mmol/L | 1.89 (0.86) | 3.04 (3.91) | 2.34 (1.61) | 2.16 (1.20) | 2.35 (2.05) |  |
| Coef.± Std. Err. (non-adjust) | Reference | 0.30 (0.08, 0.52) | 0.14 (-0.10, 0.38) | 0.07 (-0.17, 0.32) | 0.10 (-0.13, 0.33) | 0.57 |
| Coef ± Std. Err. (adjusted Model 1) | Reference | 0.31 (0.10, 0.52) | 0.17 (-0.06, 0.40) | 0.07 (-0.16, 0.31) | 0.06 (-0.16, 0.28) | 0.37 |
| Coef ± Std. Err. (adjusted Model 2) | Reference | 0.32 (0.11, 0.53) | 0.16 (-0.07, 0.39) | 0.02 (-0.21, 0.26) | 0.05 (-0.17, 0.27) | 0.23 |
| Fibrinogen^4^ |  |  |  |  |  |  |
| Participants, n | 31 | 44 | 28 | 31 | 29 |  |
| Mean, g/L | 3.12 (0.96) | 3.18 (0.98) | 2.89 (0.80) | 2.89 (0.71) | 3.47 (1.02) |  |
| Coef.± Std. Err. (non-adjust) | Reference | 0.01 (-0.11, 0.14) | -0.08 (-0.22, 0.06) | -0.06 (-0.20, 0.07) | 0.11 (-0.03, 0.25) | 0.45 |
| Coef ± Std. Err. (adjusted Model 1) | Reference | 0.03 (-0.09, 0.15) | -0.07 (-0.21, 0.06) | -0.07 (-0.20, 0.06) | 0.12 (-0.02, 0.03) | 0.52 |
| Coef ± Std. Err. (adjusted Model 2) | Reference | 0.03 (-0.11, 0.16) | -0.06 (-0.21, 0.08) | -0.07 (-0.21, 0.07) | 0.12 (-0.03, 0.26) | 0.51 |
| Homocysteine^4^ |  |  |  |  |  |  |
| Participants, n | 43 | 71 | 44 | 46 | 62 |  |
| Mean, mmol/L | 12.43 (4.45) | 11.40 (3.45) | 12.40 (6.57) | 10.94 (3.10) | 11.63 (6.48) |  |
| Coef.± Std. Err. (non-adjust) | Reference | -1.03 (-2.93, 0.87) | -0.02 (-2.13, 2.09) | -1.49 (-3.57, 0.60) | -0.79 (-2.74, 1.16) | 0.47 |
| Coef ± Std. Err. (adjusted Model 1) | Reference | -0.83 (-2.72, 1.06) | -0.42 (-2.52, 1.69) | -1.78 (-3.90, 0.33) | -1.07 (-3.08, 0.94) | 0.23 |
| Coef ± Std. Err. (adjusted Model 2) | Reference | -0.74 (-2.68, 1.20) | -0.08 (-2.24, 2.08) | -1.47 (-3.66, 0.72) | -1.04 (-3.07, 1.00) | 0.26 |
| C-reactive protein^4^ |  |  |  |  |  |  |
| Participants, n | 28 | 53 | 32 | 27 | 42 |  |
| Mean, mg/L | 2.69 (2.64) | 3.04 (4.34) | 2.63 (1.70) | 1.98 (1.58) | 4.94 (5.90) |  |
| Coef.± Std. Err. (non-adjust) | Reference | -0.05 (-0.48, 0.38) | 0.05 (-0.43, 0.53) | -0.25 (-0.75, 0.53) | 0.31 (-0.14, 0.76) | 0.22 |
| Coef ± Std. Err. (adjusted Model 1) | Reference | -0.03 (-0.46, 0.40) | 0.03 (-0.44, 0.50) | -0.37 (-0.87, 0.13) | 0.24 (-0.22, 0.69) | 0.54 |
| Coef ± Std. Err. (adjusted Model 2) | Reference | 0.05 (-0.40, 0.50) | 0.07 (-0.42, 0.56) | -0.34 (-0.87, 0.18) | 0.24 (-0.24, 0.71) | 0.67 |
| Systolic blood pressure^4^ |  |  |  |  |  |  |
| Participants, n | 45 | 70 | 45 | 47 | 61 |  |
| Mean, mmHg | 156.4 (25.3) | 154.9 (20.2) | 153.1 (26.7) | 156.5 (25.4) | 152.8 (25.3) |  |
| Coef.± Std. Err. (non-adjust) | Reference | -0.01 (-0.06, 0.05) | -0.02 (-0.096, 0.04) | 0.00 (-0.06, 0.07) | -0.02 (-0.08, 0.04) | 0.53 |
| Coef ± Std. Err. (adjusted Model 1) | Reference | -0.01 (-0.07, 0.04) | -0.02 (-0.09, 0.04) | 0.01 (-0.06, 0.07) | -0.04 (-0.10, 0.02) | 0.36 |
| Coef ± Std. Err. (adjusted Model 2) | Reference | -0.01 (-0.07, 0.05) | -0.01 (-0.07, 0.06) | 0.02 (-0.05, 0.09) | -0.04 (-0.10, 0.03) | 0.47 |
| Diastolic blood pressure |  |  |  |  |  |  |
| Participants, n | 45 | 70 | 45 | 47 | 61 |  |
| Mean, mmHg | 87.7 (10.4) | 87.4 (11.4) | 87.6 (14.7) | 86.1 (14.5) | 86.7 (13.8) |  |
| Coef.± Std. Err. (non-adjust) | Reference | -0.32 (-5.20, 4.56) | -0.09 (-5.48, 5.30) | -1.54 (-6.87, 3.79) | -0.97 (-5.99, 4.05) | 0.59 |
| Coef ± Std. Err. (adjusted Model 1) | Reference | -0.87 (-5.66, 3.92) | -0.56 (-5.86, 4.75) | -0.99 (-6.33, 4.35) | -2.78 (-7.89, 2.34) | 0.31 |
| Coef ± Std. Err. (adjusted Model 2) | Reference | 0.08 (-4.87, 5.03) | 1.09 (-4.42, 6.59) | 0.36 (-5.20, 5.93) | -2.05 (-7.26, 3.17) | 0.43 |

^1^ All values are mean ± SD; Impaired glucose tolerance, i.e. fasting glucose ≥ 6.1 mmol/L.

^2^ *P*-trend was assessed by linear regression (continuous variables) or by logistic regression (categorical variables), adjusted for age (continuous), BMI (continuous), total energy intake (continuous), alcohol consumption (quartiles), smoking (never, past or current), energy expenditure (quartiles), social class (manual or non-manual), family history of myocardial infarction (yes or no).

^3^Adjusted as model 1 plus sugar intake (<50, 50-100, >100 g/d), fruit consumption (<7, 8-14, 15-21, or >21 times/wk), red meat consumption (<7, 8-14, 15-21, or >21 times/wk) and fibre (cereal and vegetable sources) (<10, 10-20, or >20 g/d).

^4^ Original data were transformed to natural logarithms for regression model.

**Supplemental Table 3** Cross-sectional (Phase 3) analysis of metabolic markers across egg consumption categories of all subjects in Caerphilly Prospective Cohort Study^1^

|  | Egg consumption (n, eggs/wk) | | | | |  |
| --- | --- | --- | --- | --- | --- | --- |
| Characteristics | (0≤ n ≤1) | (1< n ≤2) | (2< n ≤3) | (3< n <5) | (n ≥5) | *P*-trend |
| Glucose^4^ |  |  |  |  |  |  |
| Participants, n | 450 | 505 | 301 | 230 | 229 |  |
| Fasting glucose, mmol/L | 5.62 (1.48) | 5.59 (1.45) | 5.75 (1.77) | 5.82 (1.98) | 6.09 (2.17) |  |
| Coef.± Std. Err. (non-adjust) | Reference | -0.00 (-0.03, 0.02) | 0.01 (-0.02, 0.05) | 0.02 (-0.01, 0.05) | 0.06 (-0.01, 0.05) | <0.001 |
| Coef ± Std. Err. (adjusted Model 1)^2^ | Reference | -0.01 (-0.03, 0.02) | 0.01 (-0.02, 0.04) | 0.01 (-0.03, 0.04) | 0.04 (0.01, 0.08) | 0.03 |
| Coef ± Std. Err. (adjusted Model 2)^3^ | Reference | -0.01 (-0.03, 0.02) | 0.01 (-0.02, 0.04) | 0.01 (-0.03, 0.04) | 0.04 (0.00, 0.07) | 0.04 |
| Total cholesterol |  |  |  |  |  |  |
| Participants, n | 453 | 503 | 303 | 267 | 192 |  |
| Mean, mmol/L | 6.30 (1.24) | 6.17 (1.17) | 6.25 (1.17) | 6.31 (1.04) | 6.26 (1.14) |  |
| Coef.± Std. Err. (non-adjust) | Reference | -0.13 (-0.27, 0.02) | -0.05 (-0.22, 0.11) | 0.01 (-0.17, 0.19) | -0.04 (-0.22, 0.14) | 0.85 |
| Coef ± Std. Err. (adjusted Model 1) | Reference | -0.18 (-0.35, -0.02) | -0.08 (-0.28, 0.11) | 0.05 (-0.16, 0.26) | -0.07 (-0.29, 0.15) | 0.72 |
| Coef ± Std. Err. (adjusted Model 2) | Reference | -0.20 (-0.37, 0.03) | -0.09 (-0.29, 0.10) | 0.04 (-0.17, 0.25) | -0.09 (-0.31, 0.13) | 0.84 |
| Triglycerides^4^ |  |  |  |  |  |  |
| Participants, n | 453 | 503 | 303 | 229 | 230 |  |
| Mean, mmol/L | 1.98 (1.23) | 1.84 (1.08) | 1.86 (1.08) | 1.90 (1.15) | 2.02 (1.43) |  |
| Coef.± Std. Err. (non-adjust) | Reference | -0.07 (-0.13, -0.00) | -0.05 (-0.13, 0.02) | -0.05 (-0.13, 0.04) | -0.02 (-0.10, 0.07) | 0.71 |
| Coef ± Std. Err. (adjusted Model 1) | Reference | -0.09 (-0.17, -0.02) | -0.09 (-0.17, -0.00) | -0.11 (-0.20, -0.02) | -0.09 (-0.19, 0.01) | 0.05 |
| Coef ± Std. Err. (adjusted Model 2) | Reference | -0.10 (-0.17, -0.03) | -0.09 (-0.18, -0.01) | -0.12 (-0.21, -0.03) | -0.10 (-0.19, -0.00) | 0.05 |
| Fibrinogen^4^ |  |  |  |  |  |  |
| Participants, n | 444 | 498 | 302 | 227 | 227 |  |
| Mean, g/L | 4.17 (0.95) | 4.24 (0.80) | 4.24 (0.80) | 4.21 (0.92) | 4.13 (0.83) |  |
| Coef.± Std. Err. (non-adjust) | Reference | 0.03 (0.00, 0.05) | 0.05 (0.02, 0.08) | 0.04 (0.01, 0.07) | 0.02 (-0.01, 0.06) | 0.03 |
| Coef ± Std. Err. (adjusted Model 1) | Reference | 0.01 (-0.02, 0.04) | 0.04 (0.00, 0.07) | 0.03 (-0.01, 0.06) | 0.02 (-0.02, 0.06) | 0.18 |
| Coef ± Std. Err. (adjusted Model 2) | Reference | 0.01 (-0.02, 0.04) | 0.04 (0.00, 0.07) | 0.03 (-0.01, 0.06) | 0.02 (-0.02, 0.06) | 0.17 |
| Systolic blood pressure |  |  |  |  |  |  |
| Participants, n | 466 | 520 | 313 | 238 | 236 |  |
| Mean, mmHg | 144.2 (21.9) | 142.5 (20.8) | 144.4 (23.2) | 146.7 (20.4) | 147.0 (23.8) |  |
| Coef.± Std. Err. (non-adjust) | Reference | -1.73 (-4.48, 1.01) | 0.19 (-2.95, 3.33) | 2.47 (-0.95, 5.90) | 2.85 (-0.59, 6.28) | 0.02 |
| Coef ± Std. Err. (adjusted Model 1) | Reference | 2.12 (-5.21, 0.97) | 0.01 (-3.59, 3.60) | 1.34 (-2.51, 5.19) | 2.47 (-1.59, 6.53) | 0.08 |
| Coef ± Std. Err. (adjusted Model 2) | Reference | -1.99 (-5.11, 1.12) | 0.10 (-3.52, 3.72) | 1.46 (-2.41, 5.34) | 2.44 (-1.65, 6.53) | 0.08 |
| Diastolic blood pressure |  |  |  |  |  |  |
| Participants, n | 466 | 520 | 313 | 238 | 236 |  |
| Mean, mmHg | 81.1 (11.8) | 81.5 (12.1) | 80.7 (12.2) | 81.38 (12.0) | 82.5 (11.9) |  |
| Coef.± Std. Err. (non-adjust) | Reference | 0.43 (-1.07, 1.93) | -0.36 (-2.08, 1.36) | 0.29 (-1.58, 2.17) | 1.43 (-0.45, 3.31) | 0.28 |
| Coef ± Std. Err. (adjusted Model 1) | Reference | 0.15 (-1.55, 1.84) | -0.46 (-2.43, 1.52) | -0.25 (-2.36, 1.86) | 1.17 (-1.06, 3.40) | 0.57 |
| Coef ± Std. Err. (adjusted Model 2) | Reference | 0.30 (-1.41, 2.01) | -0.38 (-2.37, 1.61) | -0.15 (-2.27, 1.98) | 1.28 (-0.97, 3.53) | 0.52 |

^1^ All values are mean ± SD.

^2^ *P*-trend was assessed by linear regression (continuous variables) or by logistic regression (categorical variables), adjusted for age (continuous), BMI (continuous), total energy intake (continuous), alcohol consumption (quartiles), smoking (never, past or current), energy expenditure (quartiles), social class (manual or non-manual), family history of myocardial infarction (yes or no), diabetes mellitus (yes or no).

^3^ Adjustd as model 1 plus sugar intake (<50, 50-100, >100 g/d), fruit consumption (<7, 8-14, 15-21, or >21 times/wk), red meat consumption (<7, 8-14, 15-21, or >21 times/wk) and fibre (cereal and vegetable sources) (<10, 10-20, or >20 g/d).

^4^ Original data were transformed to natural logarithms for regression model.

**Supplemental Table 4** Cross-sectional (Phase 3) analysis of metabolic markers across egg consumption in subjects with type 2 diabetes and/or impaired glucose tolerance from the Caerphilly Prospective Cohort study^1^

|  | Egg consumption (n, eggs/wk) | | | | |  |
| --- | --- | --- | --- | --- | --- | --- |
| Characteristics | (0≤ n ≤1) | (1< n ≤2) | (2< n ≤3) | (3< n <5) | (n ≥5) | *P*-trend |
| Glucose4 |  |  |  |  |  |  |
| Participants, n | 76 | 91 | 61 | 44 | 62 |  |
| Fasting glucose, mmol/L | 7.74 (2.60) | 7.54 (2.49) | 8.20 (2.68) | 8.51 (3.25) | 8.46 (3.05) |  |
| Coef.± Std. Err. (non-adjust) | Reference | -0.02 (-0.11, 0.06) | 0.06 (-0.04, 0.15) | 0.08 (-0.03, 0.19) | 0.08 (-0.02, 0.17) | 0.02 |
| Coef ± Std. Err. (adjusted Model 1)^2^ | Reference | 0.01 (-0.09, 0.11) | 0.09 (-0.02, 0.21) | 0.13 (-0.00, 0.26) | 0.11 (-0.01, 0.23) | 0.02 |
| Coef ± Std. Err. (adjusted Model 2)^3^ | Reference | 0.01 (-0.10, 0.12) | 0.09 (-0.03, 0.21) | 0.13 (-0.01, 0.27) | 0.09 (-0.03, 0.22) | 0.04 |
| Total cholesterol |  |  |  |  |  |  |
| Participants, n | 76 | 90 | 61 | 44 | 62 |  |
| Mean, mmol/L | 6.31 (1.13) | 6.19 (1.08) | 6.15 (1.19) | 6.19 (1.19) | 6.13 (1.00) |  |
| Coef.± Std. Err. (non-adjust) | Reference | -0.12 (-0.46, 0.22) | -0.15 (-0.53, 0.22) | -0.12 (-0.53, 0.30) | -0.18 (-0.55, 0.20) | 0.40 |
| Coef ± Std. Err. (adjusted Model 1) | Reference | -0.17 (-0.57, 0.24) | -0.14 (-0.59, 0.31) | 0.17 (-0.34, 0.68) | -0.03 (-0.50, 0.44) | 0.67 |
| Coef ± Std. Err. (adjusted Model 2) | Reference | -0.25 (-0.68, 0.18) | -0.19 (-0.67, 0.28) | 0.10 (-0.44, 0.65) | -0.08 (-0.57, 0.41) | 0.76 |
| Triglycerides^4^ |  |  |  |  |  |  |
| Participants, n | 76 | 90 | 61 | 44 | 62 |  |
| Mean, mmol/L | 2.35 (1.53) | 2.06 (1.42) | 2.08 (0.97) | 2.66 (1.71) | 2.18 (1.45) |  |
| Coef.± Std. Err. (non-adjust) | Reference | -0.12 (-0.29, 0.05) | -0.05 (-0.24, 0.13) | 0.11 (-0.09, 0.32) | -0.09 (-0.28, 0.10) | 0.92 |
| Coef ± Std. Err. (adjusted Model 1) | Reference | -0.08 (-0.28, 0.11) | -0.00 (-0.22, 0.22) | 0.10 (-0.14, 0.35) | -0.07 (-0.29, 0.16) | 0.90 |
| Coef ± Std. Err. (adjusted Model 2) | Reference | -0.09 (-0.30, 0.12) | -0.03 (-0.26, 0.20) | 0.11 (-0.15, 0.38) | -0.07 (-0.31, 0.16) | 0.92 |
| Fibrinogen^4^ |  |  |  |  |  |  |
| Participants, n | 74 | 90 | 61 | 44 | 61 |  |
| Mean, g/L | 4.08 (0.78) | 4.20 (1.16) | 4.23 (0.78) | 4.38 (0.91) | 4.26 (0.93) |  |
| Coef.± Std. Err. (non-adjust) | Reference | 0.01 (-0.05, 0.08) | 0.04 (-0.03, 0.11) | 0.07 (-0.01, 0.15) | 0.04 (-0.03, 0.11) | 0.12 |
| Coef ± Std. Err. (adjusted Model 1) | Reference | 0.02 (-0.06, 0.09) | 0.04 (-0.05, 0.13) | 0.05 (-0.06, 0.15) | 0.04 (-0.05, 0.14) | 0.30 |
| Coef ± Std. Err. (adjusted Model 2) | Reference | 0.01 (-0.08, 0.10) | 0.04 (-0.06, 0.13) | 0.04 (-0.07, 0.15) | 0.05 (-0.05, 0.14) | 0.28 |
| Systolic blood pressure |  |  |  |  |  |  |
| Participants, n | 75 | 92 | 61 | 46 | 62 |  |
| Mean, mmHg | 118.3 (22.3) | 142.9 (20.1) | 149.3 (23.1) | 150.3 (20.7) | 151.2 (22.9) |  |
| Coef.± Std. Err. (non-adjust) | Reference | -5.43 (-12.09, 1.23) | 0.99 (-6.39, 8.37) | 1.98 (-6.04, 9.99) | 2.90 (-4.45, 10.25) | 0.11 |
| Coef ± Std. Err. (adjusted Model 1) | Reference | -3.31 (-10.91, 4.29) | 0.74 (-7.84, 9.32) | 5.69 (-3.74, 15.13) | 5.32 (-3.62, 14.25) | 0.05 |
| Coef ± Std. Err. (adjusted Model 2) | Reference | -5.35 (-13.30, 2.59) | -1.23 (-10.09, 7.62) | 3.81 (-6.15, 13.76) | 4.57 (-4.57, 13.72) | 0.07 |
| Diastolic blood pressure |  |  |  |  |  |  |
| Participants, n | 75 | 92 | 61 | 46 | 62 |  |
| Mean, mmHg | 82.2 (12.1) | 82.2 (10.3) | 83.8 (11.6) | 82.2 (12.1) | 84.2 (10.9) |  |
| Coef.± Std. Err. (non-adjust) | Reference | -0.00 (-3.47, 3.46) | 1.57 (-2.27, 5.41) | -0.03 (-4.20, 4.14) | 2.01 (-1.81, 5.83) | 0.32 |
| Coef ± Std. Err. (adjusted Model 1) | Reference | 0.59 (-3.37, 4.56) | 0.99 (-3.48, 5.46) | 1.83 (-3.09, 6.75) | 3.18 (-1.48, 7.84) | 0.15 |
| Coef ± Std. Err. (adjusted Model 2) | Reference | 0.22 (-4.01, 4.45) | 0.65 (-4.07, 5.36) | 1.57 (-3.73, 6.87) | 3.27 (-1.60, 8.14) | 0.15 |

^1^ All values are mean ± SD; Impaired glucose tolerance, i.e. fasting glucose ≥ 6.1 mmol/L.

^2^ *P*-trend was assessed by linear regression (continuous variables) or by logistic regression (categorical variables), adjusted for age (continuous), BMI (continuous), total energy intake (continuous), alcohol consumption (quartiles), smoking (never, past or current), energy expenditure (quartiles), social class (manual or non-manual), family history of myocardial infarction (yes or no).

^3^ Adjustd as model 1 plus sugar intake (<50, 50-100, >100 g/d), fruit consumption (<7, 8-14, 15-21, or >21 times/wk), red meat consumption (<7, 8-14, 15-21, or >21 times/wk) and fibre (cereal and vegetable sources) (<10, 10-20, or >20 g/d).

^4^ Original data were transformed to natural logarithms for regression model.

**Supplemental Table 5** Cross-sectional analysis of metabolic markers of adult males (19-64 y) across tertiles of egg consumption (per 100 g) from the National Diet and Nutrition Survey (2009/10-2011/12)

| Characteristics, total subjects n | Coefficient | SE | *P*-trend |
| --- | --- | --- | --- |
| Fasting glucose, n=447 |  |  |  |
| Non-adjust | 0.18 | 0.11 | 0.10 |
| Multivariate adjusted | 0.15 | 0.09 | 0.10 |
| HbA1c, n=709 |  |  |  |
| Non-adjust | 0.11 | 0.05 | 0.04 |
| Multivariate adjusted | 0.09 | 0.04 | 0.05 |
| Total cholesterol, n=724 |  |  |  |
| Non-adjust | 0.10 | 0.10 | 0.36 |
| Multivariate adjusted | 0.13 | 0.10 | 0.20 |
| Triglycerides^2^, n=724 |  |  |  |
| Non-adjust | -0.09 | 0.05 | 0.09 |
| Multivariate adjusted | -0.07 | 0.05 | 0.07 |
| HDL-cholesterol^2^, n=724 |  |  |  |
| Non-adjust | 0.02 | 0.03 | 0.43 |
| Multivariate adjusted | 0.01 | 0.03 | 0.27 |
| LDL-cholesterol, n=707 |  |  |  |
| Non-adjust | 0.11 | 0.09 | 0.25 |
| Multivariate adjusted | 0.11 | 0.09 | 0.20 |
| Diastolic blood pressure, n=743 |  |  |  |
| Non-adjust | 1.19 | 1.04 | 0.26 |
| Multivariate adjusted | 1.65 | 1.03 | 0.11 |
| Systolic blood pressure, n=743 |  |  |  |
| Non-adjust | 1.82 | 1.47 | 0.08 |
| Multivariate adjusted | 0.33 | 1.50 | 0.83 |
| Total/HDL ratio^2^, n=724 |  |  |  |
| Non-adjust | 0.00 | 0.03 | 0.93 |
| Multivariate adjusted | -0.02 | 0.03 | 0.44 |
| Pulse Pressure, n=743 |  |  |  |
| Non-adjust | 0.10 | 1.02 | 0.92 |
| Multivariate adjusted | 0.95 | 1.03 | 0.36 |
| C-reactive protein, n=724 |  |  |  |
| Non-adjust | -0.13 | 0.42 | 0.76 |
| Multivariate adjusted | -0.09 | 0.43 | 0.83 |

^1^ *P*-trend was assessed by linear regression (continuous variables) or by Pearson chi-square test (categorical variables), adjusted for age (continuous), food energy (continuous), alcohol consumption (tertiles), smoking (yes or no), sex (men or women), and incident of diabetes (yes or no).

^2^ Original data were transformed to natural logarithms for regression model.

**Supplemental Table 6** Cross-sectional analysis of metabolic markers across egg consumption (per 100 g) in subjects with subjects type 2 diabetes and/or impaired glucose tolerance^1^ from the National Diet and Nutrition Survey (2009/10-2011/12)

| Characteristics, total subjects, n | Coefficient | SE | P-trend |
| --- | --- | --- | --- |
| Fasting glucose, n=59 |  |  |  |
| Non-adjust | 0.68 | 0.33 | 0.05 |
| Multivariate adjusted | 2.84 | 1.18 | 0.05 |
| HbA1c, n=57 |  |  |  |
| Non-adjust | 0.26 | 0.15 | 0.09 |
| Multivariate adjusted | 1.19 | 0.24 | 0.003 |
| Total cholesterol, n=58 |  |  |  |
| Non-adjust | 0.01 | 0.15 | 0.96 |
| Multivariate adjusted | 0.10 | 0.44 | 0.83 |
| Triglycerides^3^, n=58 |  |  |  |
| Non-adjust | -0.08 | 0.09 | 0.37 |
| Multivariate adjusted | 0.16 | 0.25 | 0.53 |
| HDL-cholesterol^3^, n=58 |  |  |  |
| Non-adjust | 0.04 | 0.04 | 0.32 |
| Multivariate adjusted | -0.04 | 0.09 | 0.64 |
| LDL-cholesterol, n=50 |  |  |  |
| Non-adjust | 0.08 | 0.13 | 0.56 |
| Multivariate adjusted | 0.12 | 0.27 | 0.68 |
| Diastolic blood pressure, n=58 |  |  |  |
| Non-adjust | -0.76 | 1.13 | 0.50 |
| Multivariate adjusted | 2.01 | 1.45 | 0.21 |
| Systolic blood pressure, n=58 |  |  |  |
| Non-adjust | -1.61 | 1.52 | 0.29 |
| Multivariate adjusted | -0.32 | 1.52 | 0.84 |
| Total/HDL ratio^3^, n=58 |  |  |  |
| Non-adjust | -0.03 | 0.05 | 0.51 |
| Multivariate adjusted | 0.09 | 0.14 | 0.57 |
| Pulse Pressure, n=58 |  |  |  |
| Non-adjust | 0.95 | 1.28 | 0.46 |
| Multivariate adjusted | 0.79 | 0.06 | 0.47 |
| C-reactive protein, n=58 |  |  |  |
| Non-adjust | -0.33 | 0.53 | 0.53 |
| Multivariate adjusted | -0.15 | 0.59 | 0.81 |

^1^ Impaired glucose tolerance, i.e. fasting glucose ≥ 6.1 mmol/L.

^2^ *P*-trend was assessed by linear regression (continuous variables) or by Pearson chi-square test (categorical variables), adjusted for age (continuous), food energy (continuous), alcohol consumption (tertiles), smoking (yes or no), sex (men or women).

^3^ Original data were transformed to natural logarithms for regression model.
